# Delivery Parameter Variations and Early Clinical Outcomes of Volumetric Modulated Arc Therapy for 31 Prostate Cancer Patients: An Intercomparison of Three Treatment Planning Systems

**DOI:** 10.1155/2013/289809

**Published:** 2013-01-15

**Authors:** Shinichi Tsutsumi, Masako N. Hosono, Daisaku Tatsumi, Yoshitaka Miki, Yutaka Masuoka, Ryo Ogino, Kentaro Ishii, Yasuhiko Shimatani, Yukio Miki

**Affiliations:** ^1^Department of Radiology, School of Medicine, Osaka City University, 1-4-3 Asahi-machi, Abeno-ku, Osaka 545-8585, Japan; ^2^Department of Radiology, Osaka City University Hospital, 1-4-3 Asahi-machi, Abeno-ku, Osaka 545-8585, Japan; ^3^Tane Hospital, 1-12-21, Kujo-Minami, Nishi-ku, Osaka 550-0025, Japan

## Abstract

We created volumetric modulated arc therapy (VMAT) plans for 31 prostate cancer patients using one of three treatment planning systems (TPSs)—ERGO++, Monaco, or Pinnacle—and then treated those patients. A dose of 74 Gy was prescribed to the planning target volume (PTV). The rectum, bladder, and femur were chosen as organs at risk (OARs) with specified dose-volume constraints. Dose volume histograms (DVHs), the mean dose rate, the beam-on time, and early treatment outcomes were evaluated and compared. The DVHs calculated for the three TPSs were comparable. The mean dose rates and beam-on times for Ergo++, Monaco, and SmartArc were, respectively, 174.3 ± 17.7, 149.7 ± 8.4, and 185.8 ± 15.6 MU/min and 132.7 ± 8.4, 217.6 ± 13.1, and 127.5 ± 27.1 sec. During a follow-up period of 486.2 ± 289.9 days, local recurrence was not observed, but distant metastasis was observed in a single patient. Adverse events of grade 3 to grade 4 were not observed. The mean dose rate for Monaco was significantly lower than that for ERGO++ and SmartArc (*P* < 0.0001), and the beam-on time for Monaco was significantly longer than that for ERGO++ and SmartArc (*P* < 0.0001). Each TPS was successfully used for prostate VMAT planning without significant differences in early clinical outcomes despite significant TPS-specific delivery parameter variations.

## 1. Introduction

 Volumetric modulated arc therapy (VMAT) provides dose distributions comparable to those of intensity modulated radiotherapy (IMRT), along with a greatly reduced delivery time [1]. A variety of treatment planning systems (TPSs) are available for VMAT planning, each of which creates a different plan in terms of the multileaf collimator (MLC) leaf positions and the dose rate during gantry rotation with varying speeds. ERGO++ (Elekta, Milan, Italy) employs an aperture-based optimization algorithm, whereas Pinnacle (Philips, Eindhoven, The Netherlands), Monaco (Elekta, MI, USA), Eclipse (Varian Medical Systems, CA, USA), and Oncentra (Elekta/Nucletron, Utrecht, The Netherlands) employ fluence-map-based optimization algorithms. The VMAT modules built into Pinnacle and Eclipse are called SmartArc and RapidArc, respectively. Hereinafter, Pinnacle SmartArc and Eclipse RapidArc are referred to as SmartArc and RapidArc for simplicity. 

 The aperture-based optimization algorithm determines the MLC leaf positions based on the shapes of the target organ and the organs at risk (OARs) in beam's eye view (BEV). A conformal field shape is used when the target is situated in front of the OARs, whereas a conformal avoidance field shape is employed when the OARs are in front of the target. Using these field shapes, the monitor units per degree for each gantry angle interval are optimized for given dose constraints. This approach is applied only to prostate cancers and other simple cases [2].

 The fluence-map-based optimization algorithm calculates optimized fluence maps for a large set of fixed gantry angles. Subsequently, an arc sequencer algorithm converts the fluence maps for multiple fixed-angle delivery to those for arc delivery while optimizing an MLC leaf shape sequence. In some TPSs, direct aperture optimization is employed instead of the above two-stage process. Because both the MLC shapes and the beam intensities are optimized, VMAT planning has been reported for various lesions including prostate cancers, rectal cancers, head and neck cancers, and brain tumors, as well as for partial breast irradiation, craniospinal irradiation, and total marrow irradiation [3–7].

Many VMAT planning studies for prostate cancer have been reported, and these studies have demonstrated that the dose distributions for VMAT are similar to those for IMRT [8–10]. However, one of the major differences between VMAT and IMRT is the dose rate characteristics during delivery. It is known that radiobiological responses significantly vary with dose rates between 1 cGy/min and 10 cGy/min. In addition, tumor responses are dependent on the dose rate even for dose rates exceeding 100 cGy/min [11, 12]. Additionally, it has been reported that normal cells such as AGO-1522b fibroblast cells exhibit increased survival with increased delivery time [13]. In Elekta VMAT, the available dose rates are 600 MU/min, 300 MU/min, 150 MU/min, 75 MU/min, 37 MU/min, and 18 MU/min; the combination of dose rates and gantry speeds is dynamically determined by a linac controller to minimize beam-on time [14]. In contrast, step-and-shoot IMRT is normally performed with the maximum dose rate during the entire segmental delivery.

Pesce reported the initial experiences with VMAT for 45 prostate cancer patients using RapidArc with a follow-up period of 2 months [15]. To the best of our knowledge, there have been no published intercomparisons of TPSs with respect to the delivered dose rate and early clinical outcomes for VMAT. The purpose of this study was to compare TPSs with respect to the VMAT delivery parameters and early clinical outcomes for 31 prostate cancer patients treated in our facility.

## 2. Methods and Materials

### 2.1. Patient Characteristics

Thirty-one prostate cancer patients were consecutively treated with VMAT from March 2009 to July 2011.  Table 1 shows the patient statistics. Thirty-one patients were classified according to the TNM staging system, Gleason score, PSA level, risk grade, and prior hormone therapy. The number of plans created by each treatment planning system is also shown.

### 2.2. Treatment Planning

To create a single-arc VMAT plan, ERGO++, Monaco, and SmartArc were used between March 2009 and May 2010, between June 2010 and January 2011, and between February 2011 and July 2011, respectively. The patients were asked to refrain from urinating within one hour prior to the acquisition of the planning CT. This CT scan was performed in the supine position with a slice thickness of 2 mm and a Vac-Lok fixation device (CIVCO Medical Solutions, IA, United States). Pinnacle was always used to contour structures. The clinical target volume (CTV) consisted of the entire prostate and the base of the seminal vesicle, which includes the inner one-third of the lateral width and extends longitudinally to the branching point. The planning target volume (PTV) was generated by adding a 10 mm margin to the CTV in all dimensions except posteriorly, for which a 7 mm margin was used. The OARs included the rectum, the bladder, and the femoral heads. The rectum was contoured from 1 cm above to 1 cm below the PTV as a solid organ. The femoral heads were contoured inferiorly to the lesser trochanter.

 Optimization was performed in each TPS to obtain a single-arc VMAT plan. A previous article reported that a prescribed dose of 74 Gy is superior to a dose of 64 Gy in terms of the disease-free survival rate [16]. 

In addition, clinical outcomes for treatment with 72 Gy or greater are equivalent to or superior to those for surgery, whereas the outcomes for 72 Gy or less are inferior to those for surgery [17]. Based on these findings, a prescribed dose of 74 Gy was employed; however, 70 to 72 Gy was employed for the initial five patients, who did not tolerate VMAT treatment well. Furthermore, two patients received 70 Gy to minimize side effects; one of these patients suffered from Parkinson's disease, and the other was being treated after surgery for sigmoid colon cancer. The dose constraints for the PTV were as follows: maximum dose ≤ 80 Gy and *D*_99%_ ≥ 70.3 Gy (95% of the prescribed dose). The dose constraints for the rectum were as follows: maximum dose ≤ 80 Gy, *V*_70 Gy_ ≤ 15%, *V*_60 Gy_ ≤ 35%, and *V*_50 Gy_ ≤ 50%. The dose constraints for the bladder were as follows: maximum dose ≤ 80 Gy, *V*_75 Gy_ ≤ 15%, *V*_70 Gy_ ≤ 25%, and *V*_60 Gy_ ≤ 50%. The dose constraints for the each of the femoral heads were as follows: maximum dose ≤ 45 Gy. All of the OAR dose constraints were based on previous reports [18–20].

### 2.3. Plan Evaluation

For ERGO++ and SmartArc, dose volume histograms (DVHs) were calculated using a superposition algorithm implemented within the Pinnacle TPS. For Monaco, a Monte Carlo algorithm was used for the DVH calculation. 

### 2.4. Dosimetric Verification

The point doses were verified at five different points, including the isocenter, and the dose distributions were examined using films in the axial, coronal, and sagittal planes, each including the isocenter. 

### 2.5. Treatment

An Elekta linac, Synergy (Elekta, Crawley, UK), was used with a photon energy of 6 MV for ERGO++ and 10 MV for Monaco and SmartArc. The dose rates were determined by the Synergy linac controller, and a dose rate ranging between 18 MU/min and 300 MU/min was dynamically selected during VMAT delivery. As for the acquisition of the planning CT, the patients were asked to refrain from urinating within one hour prior to treatment. Then, a cone-beam CT (CBCT) scan was performed using an on-board X-ray volume imaging device, XVI (Elekta, Crawley, UK), in the supine position immediately before every treatment. Tumor registration was performed by comparing the planning CT and CBCT images, and the treatment couch was repositioned for accurate dose delivery.

### 2.6. TPS Intercomparison

The DVH parameters for the PTV and the OARs, the total MU, the beam-on time, and the mean dose rates were evaluated and compared. For PTV, the homogeneity index and conformity index were calculated, where the homogeneity index was given by (*D*_2%_ − *D*_98%_)/*D*_prescribed  dose_, and the conformity index was based on the RTOG rule [21].

### 2.7. Statistical Analyses

All statistical analyses were performed using GraphPad Prism V5.04 (GraphPad Software, San Diego, CA). The Kruskal-Wallis test was employed to identify differences in the means among the plans created by the three different TPSs using *P* values. Subsequently, Dunn's multiple comparison test was performed for the cases with *P* < 0.05 to compare the means between plans created by each pair of the three TPSs. 

### 2.8. Toxicity Evaluation

CTCAE v4.0 was applied for toxicity grading, and rectal bleeding, hematuria, and other adverse GI and GU events of grades 3 to 4 were analyzed.

## 3. Results

VMAT delivery was successfully completed for all 31 patients. The dose verification results were satisfactory in both the point and film measurements. As described earlier, seven patients received a prescribed dose between 70 and 72 Gy. A prescribed dose of 74 Gy was applied to 10 patients in the ERGO++ group, 9 patients in the Monaco group, and 5 patients in the SmartArc group, totaling 24 cases with a prescribed dose of 74 Gy. The dose comparison was performed using these 24 cases.

**Figure 1:**
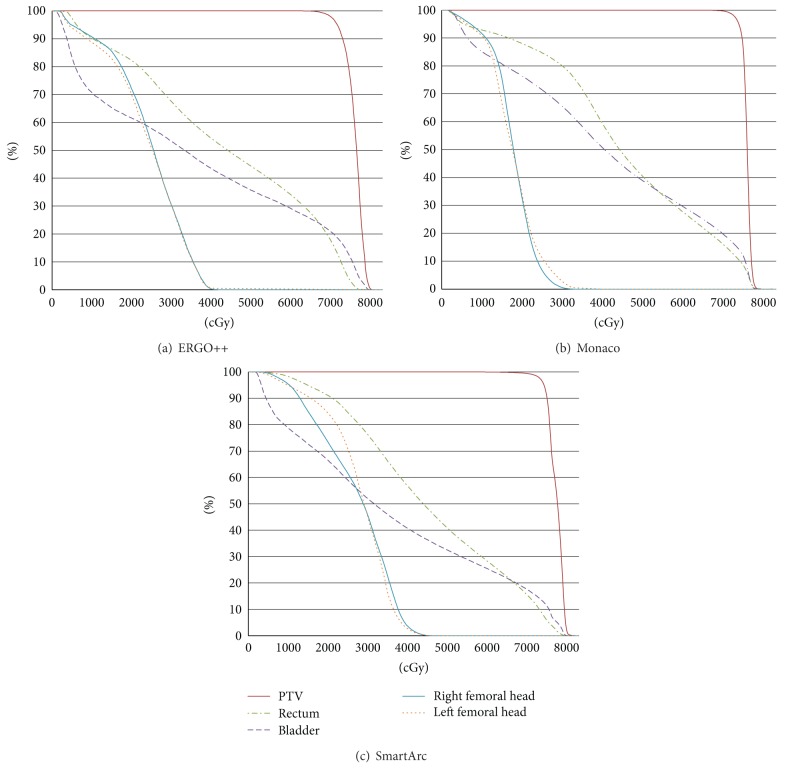
Comparison of the dose volume histogram (DVHs) for ERGO++, Monaco, and SmartArc. Each plot shows the patient average with a prescribed dose of 74 Gy.

Figure 1 shows the DVH comparisons for the ERGO++, Monaco, and SmartArc groups. Each plot shows the patient average with a prescribed dose of 74 Gy. The DVHs calculated by the three TPSs were comparable.

Table 2 shows the comparison of the plans created by ERGO++, Monaco, and SmartArc in terms of the total MU, the beam-on time, the mean dose rate during delivery, and the DVH parameters of the PTV and OARs. The symbols + and − indicate that the difference is significant and insignificant, respectively, with a threshold probability of 5%.

 In the PTV, *D*_95%_ and *D*_98%_ for ERGO++ were significantly lower than those for Monaco and SmartArc (*P* = 0.0043), whereas there was no significant difference in *D*_2%_ among the three TPSs. The conformity index and homogeneity index for ERGO++ were significantly lower than those for Monaco and SmartArc. The doses for the rectum, bladder, and femoral heads were comparable and met all of the given constraints with slight significance in the differences for some parameters.

**Figure 2:**
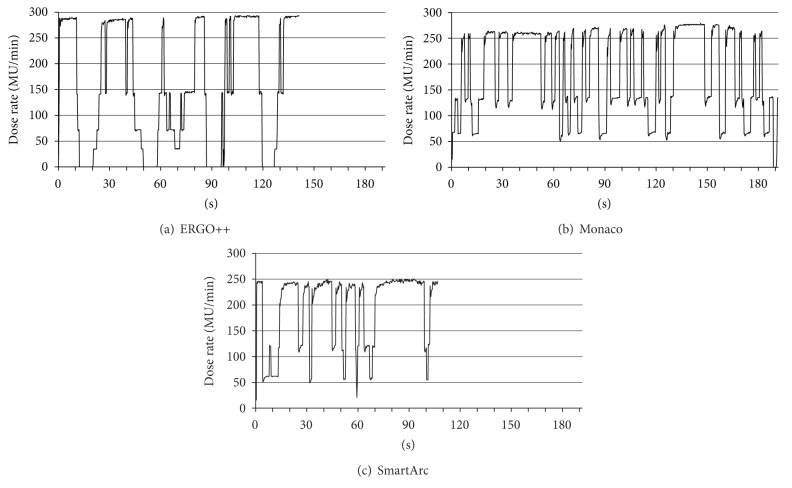
Plots of typical dose rate variations as a function of time during VMAT delivery. The plans were created with (a) ERGO++, (b) Monaco, and (c) SmartArc.

For delivery parameters, the total MUs for ERGO++, Monaco, and SmartArc were 383.7 ± 27.3, 541.8 ± 26.9, and 395.5 ± 97.3, respectively; the total MUs for Monaco were significantly higher than those for ERGO++ and SmartArc (*P* < 0.0001). The beam-on times for ERGO++, Monaco, and SmartArc were 132.7 ± 8.4 sec, 217.6 ± 13.1 sec, and 127.5  ±  27.1 sec, respectively; the beam-on times for ERGO++ and SmartArc were significantly shorter (*P* < 0.0001) than that for Monaco. The mean dose rate during VMAT delivery for Monaco was significantly higher than those for ERGO++ and SmartArc; however, the variation ranged between 150 and 200 MU/min.  Figure 2 shows plots of the typical dose rate variations as a function of time during VMAT delivery. The plans were created using (a) ERGO++, (b) Monaco, and (c) SmartArc. 

During a follow-up period of 486.2 ± 289.9 days, local recurrence was not observed, but distant metastasis in the form of multiple bone metastasis was observed in a single patient on the 176th day after the treatment was completed.  Table 3 shows the early treatment toxicities. Grade 1 rectal bleeding occurred in four out of the 31 patients (two in ERGO++ and two in Monaco). Hematuria and other adverse GI and GU events of grades 3 to 4 were not observed. The symptoms disappeared shortly without treatment while monitoring the patients. Possible causes of the lack of rectal bleeding with SmartArc may include the shorter follow-up period or variations in patient-specific factors.

## 4. Discussion

We showed in this study that the physical parameters vary during VMAT delivery when using different TPSs due to the different optimization algorithms employed, even when using nearly equivalent dose constraints for the PTV and the OARs. The impact of the delivery parameter variations on clinical outcome may need to be addressed further with a longer follow-up period. 

In this study, the DVH parameters were all within the planned constraints and were thus satisfactory. The *D*_95%_ of the PTV dose for ERGO++ was significantly less than that for Monaco and SmartArc. There are two causes for this reduction: (1) a dose of 74 Gy was prescribed to the isocenter, not *D*_95%_, and (2) ERGO++ uses a pencil beam algorithm for dose calculation, which is known to be less accurate than the superposition algorithm employed in the Pinnacle TPS [22]. However, the dose reduction was considered clinically acceptable. The DVH variations for the OARs among three TPSs were relatively small and insignificant. Therefore, the three TPSs were practically equivalent for the planning of VMAT for the treatment of prostate cancer. 

We showed that there are variations in the total MUs and beam-on times among the three TPSs; however, the total MUs and the beam-on times for the VMAT plans are much lower than those for IMRT, thus suggesting that VMAT delivery is superior [8]. The mean dose rates were less than 200 MU/min for the three TPSs. Specifically, ERGO++ resulted in a longer delivery period with a higher dose rate of nearly 300 MU/min while providing a period in which the gantry was moved without beam delivery. Monaco did not generate this type of move-only period in practice while leading to a longer delivery period with lower dose rates ranging between 50 and 150 MU/min. SmartArc resulted in a shorter delivery period with the lower dose rates (50 to 150 MU/min) and a shorter move-only period. As shown in Table 2, SmartArc provided the largest mean dose rate among the three TPSs.

Distant metastasis was observed in a single patient, and local tumor control was observed in all the cases; therefore, we could not find any difference in the early treatment outcomes among the three TPS groups. The toxicities were also comparably satisfactory, and the early clinical outcomes and toxicities are nearly equivalent and comparable to the results presented in a previous report on IMRT [23]. 

One limitation of the current study is the unknown variations in the patient population. However, the prostate gland is not considered to exhibit large anatomical variations among patients, thereby allowing us to perform an intercomparison of the different TPSs. Another concern is the different photon energies employed by the three TPSs. 

In conclusion, the three TPSs provided virtually equivalent early clinical outcomes and toxicities even though they exhibited significant TPS-specific delivery parameter variations.

Late outcomes and toxicities need to be studied with a longer follow-up period.

## Figures and Tables

**Table 1:** Patient characteristics. Thirty-one patients were classified according to the TNM staging system, Gleason score, PSA level, risk grade, and prior hormone therapy. The number of plans created by each treatment planning system is also shown.

	Total	ERGO++	Monaco	SmartArc
T stage				
1c	15	8	4	3
2a	7	2	3	2
2b	7	3	3	1
2c	1	1	0	0
3b	1	1	0	0
N stage				
0	31	15	10	6
M stage				
0	31	15	10	6
Gleason score				
6	8	4	3	1
7	16	8	5	3
8	7	3	2	2
PSA level				
0–10 ng/dL	18	9	5	4
10–20 ng/dL	11	5	4	2
>20 ng/dL	2	1	1	0
Risk grade				
Low	6	3	2	1
Intermediate	17	9	5	3
High	8	3	3	2
Hormone therapy				
(+)	20	10	7	3
(−)	11	5	3	3

**Table 2:** Comparison of plans created by ERGO++, Monaco, and SmartArc in terms of the DVH parameters for the PTV and OARs, the total MUs, the beam-on time, and the mean dose rate during delivery. For each category, the Kruskal-Wallis test was employed to identify differences in the means among plans created by the three different TPSs using *P* values. Subsequently, Dunn's multiple comparison test was performed for the cases with *P* < 0.05 to compare the means between plans created by each pair of the three TPSs. The symbols + and − indicate that the difference is significant and insignificant, respectively, with a threshold probability of 5%.

	ERGO++	Monaco	SmartArc	*P* value	ERGO++ versus Monaco	ERGO++ versus SmartArc	Monaco versus SmartArc
PTV							
*D*_95%_ (Gy)	72.1 ± 1.5	73.8 ± 0.2	74.3 ± 0.2	<0.01	+	+	−
*D*_98%_ (Gy)	70.5 ± 1.7	72.5 ± 0.5	72.6 ± 1.6	<0.01	+	+	−
*D*_2%_ (Gy)	78.6 ± 1.2	77.4 ± 0.4	78.7 ± 1.8	0.04	−	−	−
Conformity index	0.84 ± 0.14	0.94 ± 0.01	0.96 ± 0.01	<0.01	−	+	−
Homogeneity index	0.11 ± 0.02	0.07 ± 0.01	0.08 ± 0.04	<0.01	+	−	−
Rectum							
*D*_max⁡_ (Gy)	76.9 ± 1.1	78.5 ± 0.5	78.1 ± 1.5	<0.01	+	−	−
*V*_75 Gy_ (%)	3.1 ± 3.1	8.0 ± 2.5	5.7 ± 3.1	0.02	+	−	−
*V*_70 Gy_ (%)	17.6 ± 7.12	15.8 ± 4.3	15.3 ± 1.4	0.82			
*V*_60 Gy_ (%)	34.0 ± 9.10	27.4 ± 7.0	28.0 ± 3.0	0.21			
*V*_50 Gy_ (%)	43.3 ± 10.6	40.1 ± 9.6	40.4 ± 4.7	0.77			
Bladder							
*D*_max⁡_ (Gy)	78.7 ± 1.3	78.1 ± 0.5	78.8 ± 1.8	0.37			
*V*_75 Gy_ (%)	11.4 ± 6.4	11.5 ± 4.0	10.8 ± 6.6	0.79			
*V*_70 Gy_ (%)	20.9 ± 8.6	19.6 ± 6.4	17.9 ± 8.8	0.79			
*V*_60 Gy_ (%)	29.1 ± 12.5	29.5 ± 10.0	26.0 ± 12.0	0.75			
*V*_50 Gy_ (%)	35.7 ± 14.9	38.6 ± 13.0	33.2 ± 15.1	0.68			
Right femoral head							
*D*_max⁡_ (Gy)	39.9 ± 3.1	29.5 ± 3.9	41.9 ± 5.4	<0.01	+	−	+
Left femoral head							
*D*_max⁡_ (Gy)	40.3 ± 2.8	30.7 ± 4.0	42.6 ± 3.5	<0.01	+	−	+
MU	383.7 ± 27.3	541.8 ± 26.9	395.5 ± 97.3	<0.01	+	−	+
Beam-on time (second)	132.7 ± 8.4	217.6 ± 13.1	127.5 ± 27.1	<0.01	+	−	+
Dose rate (MU/min)	174.3 ± 17.7	149.7 ± 8.4	185.8 ± 15.6	<0.01	+	−	+

**Table 3:** Early treatment toxicities. Grade 1 rectal bleeding occurred in four out of the 31 patients. Hematuria and other adverse GI and GU adverse of grades 3 to 4 were not observed.

	Total	ERGO++ (74 Gy)	Monaco (74 Gy)	SmartArc (74 Gy)
Rectal breeding				
			
G1	4	2	2	0
G2, 3, 4	0	0	0	0
Other GI				
G3, 4	0	0	0	0
Hematuria				
G1, 2, 3, 4	0	0	0	0
Other GU				
G3, 4	0	0	0	0
